# The relative survival and cure fraction of gastric cancer estimated through flexible parametric models using data from population‐based cancer registration during 2003‐2012 in Linzhou, China

**DOI:** 10.1002/cam4.2831

**Published:** 2020-01-28

**Authors:** Qiong Chen, Shu‐Zheng Liu, Shao‐kai Zhang, Xiao‐Qin Cao, Bian‐Yun Li, Pei‐Liang Quan, Lan‐Wei Guo, Lee Dong, Xi‐Bin Sun, Yawei Zhang, Jian‐Gong Zhang

**Affiliations:** ^1^ Department of Epidemiology Affiliated Cancer Hospital of Zhengzhou University/ Henan Provincial Cancer Hospital Zhengzhou China; ^2^ Linzhou Cancer Registry Linzhou Cancer Hospital Linzhou China; ^3^ University of Chicago Chicago IL USA; ^4^ Department of Surgery Yale University School of Medicine New Haven CT USA; ^5^ Department of Environmental Health Sciences Yale School of Public Health New Haven CT USA

**Keywords:** cure fraction, cure model, Gastric cancer, relative survival

## Abstract

**Purpose:**

The proportion of cured gastric cancer patients has drawn the attention of patients, physicians, and healthcare providers after comprehensive prevention and control measures were carried out for several years. Therefore, the relative survival and cure fraction were estimated in our study.

**Methods:**

Population‐based cancer registration data were used to estimate survival and cure fraction. A total of 7585 gastric cancer cases (ICD10:C16.0 ~ C16.9) were extracted and included in the final analysis. Cases were diagnosed in 2003‐2012 and followed until the end of 2017. Relative survival was calculated as the ratio between the observed survival through the life‐table method. The expected survival was estimated by the Ederer II method. The cure fraction was estimated using flexible parametric cure models stratified by age and calendar period when the cases were diagnosed.

**Results:**

The 5‐year relative survival of cardia gastric cancer increased with the calendar period of 2003‐2004, 2005‐2006, 2007‐2008, 2009‐2010, and 2011‐2012 (27.5%, 28.3%, 33.5%, 38.2%, and 46.8%, respectively). The increasing trend along with the calendar periods was also observed in cure proportion of cardia gastric cancer (24.8%, 25.2%, 31.7%, 36.0%, and 43.1%, respectively). Notable improvement of cure proportion was observed in the period of 2011‐2012, compared with the initial period of 2003‐2004. There was an improvement of 79.8% among all gastric cancer subjects, and it was 74.1% and 55.7% in cardia gastric and noncardia gastric cancer subjects, respectively. The median survival of “uncured” patients showed no significant improvement along with the calendar periods in all age groups.

**Conclusions:**

Notable improvement of gastric cancer relative survival and cure proportion was observed in Linzhou during 2003‐2012.

## Graphical abstract

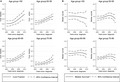

## 1 INTRODUCTION

The vast geographical difference in gastric cancer incidence and mortality existed worldwide; China is among the high‐risk areas in the world.^1^ The gastric cancer occurrence also differs largely in different areas of China; Linzhou, which lies in the east side of the Taihang Mountains with a population of 1.1 million, was known for high incidence of esophageal and gastric cancer in the world.^2^, ^3^ Due to the large burden of gastric cancer, an organized gastric cancer screening program supported by the national fund was carried out in the population beginning in 2005 to increase the gastric cancer survival, with the ultimate purpose of decreasing the incidence and mortality.^3^

Five‐year gastric cancer survival rates are at 90% for early‐stage patients, but only about 10% for advanced‐stage patients.^4^ Early‐stage gastric cancer and precancer are difficult to diagnose in routine medical services due to lack of symptoms in patients; thus, patients were usually found in advanced stage, with low prognoses. The implementation of organized gastric cancer screening programs would increase the proportion of early cancer and precancerous diagnoses. These patients can be advised to be treated with appropriate methods by the screening guideline, decreasing the incidence, and increasing the survival of gastric cancer in screening areas. The effect of gastric cancer screening was evaluated in previous studies: 4.5% of screening population were detected with lesions of low‐grade intraepithelial neoplasia and above, and 0.2% were diagnosed with early cancer in Linzhou.^5^ It was also evaluated in several studies in China,^3^ Korea,^6^ and Japan,^7^, ^8^ and the risk of death from gastric cancer was reduced by around 30%‐60%. The effect of endoscopic screening would be affected by several factors, such as sample size and screening rate, in which the skills of endoscopist and availability of gastroscope were thought to be pivotal. In the previous study, the gastric cancer relative survival was also evaluated; it was shown to increase each year and was even higher than the national average relative survival.^9^ However, the evidence that evaluates the organized gastric cancer screening effect remains deficient.

The gastric cancer intervention was evaluated by varied indexes including proportion of precancerous lesions detected and treated, survival, and risk of death.^3^, ^5^, ^9^ However, healthcare providers, physicians, and patients still believe that the proportion of cured patients by varied interventions and advanced treatment needs further research. This value could be determined by introducing the concept of “statistical cure,” which happens when the curve of cumulative relative survival shows a plateau; the cure fraction then can be defined as the proportion of cured patients in the plateau out of all patients.^10^, ^11^, ^12^, ^13^

In recent studies, cure fraction and survival function were estimated using cure models, in which the cancer patients were divided into uncured patients and cured patients. In this study, cured patients are defined as having the same mortality rate with people of the same age and sex in the general population; thus, cured patients will not experience excess mortality due to the cancer.^13^, ^14^, ^15^ Flexible parametric models were seen to fit better than the traditional mixture or nonmixture cure models^10^, ^16^, ^17^; therefore, relative survival and cure fraction of gastric cancer were estimated using population‐based cancer registration data through the flexible parametric cure models.

## 2 MATERIALS AND METHODS

### 2.1 Data source and quality control

The subjects included in our study were extracted from the database of Linzhou Cancer Registry, which is one of the earliest Cancer Registries in China and was established in the 1960s. The procedures of data collection, standards of cancer cases coding, and the validity check of cancer cases were conducted according to the criteria of “International Agency for Research on Cancer/International Association of Cancer Registries (IARC/IACR)” and “Guideline for Chinese Cancer Registration.” The cancer cases were sourced using records of hospitals, health insurance system, new rural cooperative medical systems, and vital statistical systems.^18^ The quality control procedures had been described previously, they would be summarized then as follows.^18^ The quality control indices of the Linzhou cancer registration data including comparability, validity, and completeness were evaluated by the IARC/IACR and the National Central Cancer Registry of China (NCCR). The data were included in the National Cancer Registry Annual Report in China, and the data during 2008‐2012 were accepted and included by the IARC/IACR publications (Cancer Incidence in Five Continents, Vol. XI).^19^

### 2.2 Follow‐up procedure

Follow‐up procedures were conducted using the methods of passive follow‐up and active follow‐up, which were carried out by cancer registrars and village doctors, respectively. Firstly, the cancer registration database was routinely linked to the vital statistic database to obtain the follow‐up information including cause of death, date of death, and place of death. The procedure was usually conducted once each quarter of a year. Secondly, the unmatched cancer patients would be followed up actively and annually through the methods including telephone and home visiting. All gastric cancer cases included in our study were followed up until the emergence of outcome events: the death, emigration, or the end of the study on July 1, 2018, and 0.2% of the subjects were lost to follow‐up.

### 2.3 Subjects

In our study, the subjects were extracted from the Linzhou cancer registration database based on ICDO3 code. The records in which primary site in C16.0‐16.9 and behavior was malignant were included. The data quality indicators including the mortality‐to‐incidence (M/I) ratio, the percentage of cases morphologically verified (MV) (%), and the percentage of death certificate‐only cases (DCO) (%) were used to evaluate the validity, reliability, and completeness of cancer registration, and they were 0.8, 90.0%, and 0.9%, respectively. All gastric cancer cases diagnosed during the calendar year between 2003 and 2012 in the database were included in the analysis. All cancer subjects in the cancer registration database that were sourced based on only death certificates were excluded in our study. In total, 7585 gastric cancer subjects were included in the final analysis, and 25.0% of the subjects were censored cases.

### 2.4 Statistical analysis

The age‐standardized incidence and mortality were calculated using Segi's standardized population. The relative survival was calculated as the ratio between the observed survival through the life‐table method. The expected survival was estimated by the Ederer II method.^20^ Cure fraction and survival function were estimated using flexible parametric model in which the point of statistical cure happens when the curve of cumulative relative survival shows a plateau, and the proportion at the point is defined as the estimated cure fraction. Confidence intervals were estimated by the application of the delta method.^21^ And instead of using a specific parametric distribution, the shape of the survival distribution was modeled with restricted cubic spline with 4 internal knots at 20th, 40th, 60th, and 80th centiles on the distribution of uncensored log event times.^13^, ^22^ The age of subjects was reclassified into four age groups (19‐49, 50‐59, 60‐69, and 70‐99), and calendar years were reclassified to five calendar periods (2003‐2004, 2005‐2006, 2007‐2008, 2009‐2010, and 2011‐2012) for further analysis. Calendar years were modeled continuously using splines when generating figures. The evaluation of the fit of the flexible parametric cure models was conducted by comparing their estimated relative survival with that calculated by the empirical life‐table and Ederer II method. All analysis was conducted through the stpm2 package in stata13.0.

## 3 RESULTS

Demographic characteristics are shown in Table 1. A total of 7585 gastric cancer patients (67.9% males and 32.1% females) were diagnosed in Linzhou cancer registration population, and they were included in this study. During the period of 2003‐2012, the age‐standardized incidence and mortality were 78.7 and 58.6 per hundred thousand population, respectively. The average age when the patients were diagnosed with gastric cancer was 62.1 ± 10.1 years. Among these patients, 65.2% of them occurred in the cardia gastric region, 17.4% occurred in noncardia gastric region, and 17.5% subsites were unknown. Most of the patients were aged 50 years or older, and 9.7% were aged younger than 50 years.

**Table 1:** Demographic characteristics of patients diagnosed with gastric cancer in Linzhou during 2003‐2012

Calendar periods	2003‐2004	2005‐2006	2007‐2008	2009‐2010	2011‐2012	Total
Person years	1 971 634	2 020 137	2 041 501	2 061 977	2 136 434	10 231 683
Standardized incidence (1/10^5^)*	76.5	73.0	86.2	98.1	69.2	78.7
Standardized mortality (1/10^5^)*	59.5	60.6	61.7	66.9	52.0	58.6
**Gender, n (%)**						
Male	890 (66.8)	863 (67.0)	1005 (67.5)	1156 (68.2)	1235 (69.3)	5149 (67.9)
Female	442 (33.2)	425 (33.0)	483 (32.5)	540 (31.8)	546 (30.7)	2436 (32.1)
**Age groups, n (%)**						
19‐50 years	182 (13.7)	123 (9.6)	133 (8.9)	148 (8.7)	151 (8.5)	737 (9.7)
50‐59 years	425 (31.9)	429 (33.3)	494 (33.2)	518 (30.5)	491 (27.6)	2357 (30.1)
60‐69 years	419 (21.5)	381 (29.6)	497 (33.4)	603 (35.6)	696 (39.1)	2596 (34.2)
70‐99 years	306 (23.0)	355 (27.6)	364 (24.5)	427 (25.2)	443 (24.9)	1895 (25.0)
Mean age at diagnosis	61.0 ± 10.3	62.3 ± 10.4	62.0 ± 9.8	62.3 ± 10.2	62.9 ± 10.0	62.1 ± 10.1
**Subsites, n (%)**						
Cardia gastric	834 (62.6)	724 (56.2)	1016 (68.3)	1134 (66.9)	1237 (69.5)	4945 (65.2)
Noncardia gastric	285 (21.4)	145 (11.3)	232 (15.6)	326 (19.2)	328 (18.4)	1316 (17.4)
Subsite unknown	213 (16.0)	419 (32.5)	240 (16.1)	236 (13.9)	216 (12.1)	1324 (17.5)

* Standardized incidence and mortality were adjusted using Segi's standardized population.

The relative survival in different age groups during periods 2003‐2012 is shown in Table 2. The relative survival improved along with the calendar periods among all gastric cancer patients regardless of gastric subsites. Compared with the relative survival rate in period 2003‐2004, there was a 40.4%, 41.3%, and 28.4% improvement in total, cardia gastric, and noncardia gastric, respectively, in the period 2011‐2012. This trend was also observed in all age groups among all gastric cancer.

**Table 2:** Gastric cancer 5‐year relative survival rate (%) of different age groups stratified by anatomical subsites in Linzhou during 2003‐2012

Calendar periods	2003‐2004	2005‐2006	2007‐2008	2009‐2010	2011‐2012
**Total**					
19‐49 years	26.4	26.8	37.1	37.8	49.6
50‐59 years	29.3	28.3	37.9	40.8	46.7
60‐69 years	27.5	21.0	31.0	35.2	45.7
70‐99 years	20.0	14.9	20.4	28.4	36.7
Total	26.2	22.9	31.7	35.6	43.9
**Cardia gastric**					
19‐49 years	29.2	31.2	38.7	37.7	53.4
50‐59 years	29.4	34.8	41.5	45.1	48.4
60‐69 years	30.2	25.4	30.5	38.0	48.1
70‐99 years	18.3	21.0	22.7	28.3	41.5
Total	27.5	28.3	33.5	38.2	46.8
**Noncardia gastric**					
19‐50 years	35.9	37.9	42.5	39.4	46.5
50‐59 years	32.4	26.4	36.1	32.9	43.0
60‐69 years	24.2	21.8	30.3	32.0	38.1
70‐99 years	17.7	5.9	19.9	32.7	30.0
Total	27.9	23.7	32.3	33.1	38.9

**Figure 1:**
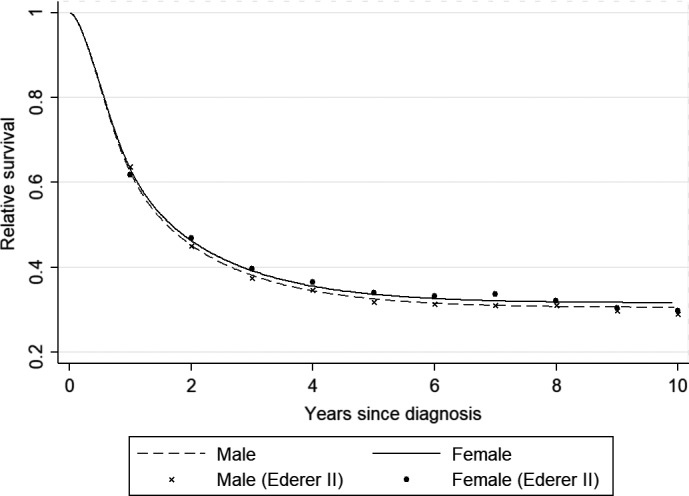
Predicted relative survival for patients in all age groups diagnosed during 2003‐2012 stratified by gender in Linzhou

As shown in Figure 1, the relative survival rates estimated by fitting flexible parametric models were in line with those calculated using the empirical life‐table and Ederer II method. This suggests that the survival curve tends to reach a plateau after 5 years.

**Figure 2:**
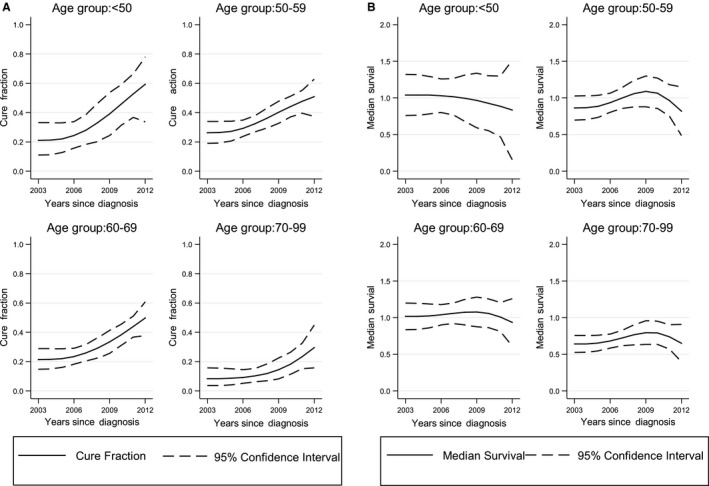
(A) Estimated gastric cancer cure fraction with 95% confidence intervals stratified by age group. (B). Median survival of the uncured group with 95% confidence intervals stratified by age group

As shown in Table 3 and Figure 2A, the cure fraction of gastric cancer improved along with the calendar years across all age groups. Among all gastric cancer subjects, the improvements of cure fraction in calendar period 2011‐2012 were 110.0%, 71.9%, 68.4%, and 143.4% in 19‐49, 50‐59, 60‐69, and 70‐99 years of age groups, and 79.8% in all age groups compared with the calendar period 2003‐2004. Among cardia gastric cancer subjects, the improvements of cure fraction in calendar period 2011‐2012 were 91.8%, 64.6%, 64.8%, and 196.7% in 19‐49, 50‐59, 60‐69, and 70‐99 years of age groups, and 74.1% in all age groups compared with the calendar period 2003‐2004. Among noncardia gastric cancer subjects, the improvements of cure fraction in calendar period 2011‐2012 were 41.7%, 68.6%, 46.6%, and 66.6% in 19‐49, 50‐59, 60‐69, and 70‐99 years of age groups, and 55.7% in all age groups compared with the calendar period 2003‐2004.

**Table 3:** Cure proportions (%) for patients diagnosed with gastric cancer in Linzhou during 2003‐2012

Year	Age group (years)				Total
	19‐49	50‐59	60‐69	70‐99	
**Total**					
2003‐2004	23.5 (17.8‐29.7)	27.6 (23.4‐32.0)	25.2 (21.0‐29.6)	11.1 (7.8‐15.0)	23.0 (20.6‐25.5)
2005‐2006	22.3 (15.7‐29.7)	25.7 (21.6‐29.9)	21.3 (17.4‐25.6)	10.3 (7.3‐13.9)	20.6 (18.2‐23.0)
2007‐2008	36.2 (28.3‐44.2)	36.6 (32.3‐41.0)	29.4 (25.2‐33.6)	15.8 (12.0‐20.1)	30.0 (27.4‐32.5)
2009‐2010	37.1 (29.5‐44.8)	41.1 (36.8‐45.4)	33.4 (29.5‐37.4)	20.3 (16.3‐24.7)	33.7 (31.2‐36.2)
2011‐2012	49.4 (41.0‐57.3)	47.4 (42.7‐52.0)	42.4 (38.4‐46.4)	27.0 (22.4‐31.8)	41.4 (38.8‐44.0)
**Cardia gastric**					
2003‐2004	26.2 (18.0‐35.1)	29.4 (24.1‐34.9)	26.8 (21.5‐32.3)	10.3 (6.3‐15.4)	24.8 (21.6‐28.0)
2005‐2006	23.1 (13.2‐34.8)	33.0 (27.1‐39.1)	25.3 (19.6‐31.1)	12.0 (7.6‐17.4)	25.2 (21.8‐28.8)
2007‐2008	37.7 (27.3‐48.1)	39.4 (34.1‐44.8)	29.3 (24.4‐34.3)	18.6 (13.5‐24.3)	31.7 (28.5‐34.9)
2009‐2010	38.2 (27.5‐48.8)	44.2 (38.8‐49.5)	36.0 (31.2‐40.9)	20.5 (15.4‐26.1)	36.0 (32.9‐39.2)
2011‐2012	50.2 (38.0‐61.2)	48.4 (42.6‐53.9)	44.1 (39.4‐48.8)	30.7 (24.7‐36.8)	43.1 (39.9‐46.3)
**Noncardia gastric**					
2003‐2004	31.9 (19.7‐44.7)	26.4 (17.4‐36.2)	24.5 (15.5‐34.6)	13.3 (6.2‐23.2)	24.4 (19.1‐30.0)
2005‐2006	35.7 (17.8‐54.1)	24.2 (13.0‐37.3)	28.4 (15.9‐42.3)	5.9 (1.2‐16.4)	24.5 (17.3‐32.3)
2007‐2008	42.1 (25.0‐58.3)	36.2 (25.9‐46.5)	28.5 (17.9‐39.9)	15.0 (6.4‐26.9)	31.3 (25.0‐37.9)
2009‐2010	37.4 (23.0‐51.7)	36.4 (27.7‐45.2)	30.6 (21.2‐40.5)	22.3 (13.1‐32.9)	32.2 (26.8‐37.7)
2011‐2012	45.2 (31.3‐58.1)	44.5 (34.5‐54.0)	35.9 (26.0‐45.8)	22.1 (12.7‐33.2)	38.0 (32.2‐43.7)

As shown in Table 4 and Figure 2B, the median survival of “uncured” patients did not change significantly along with the calendar periods in all age groups. The difference in median survival between 2003‐2004 and 2011‐2012 was 0.1 year among all gastric cancer subjects, cardia gastric cancer cases, and noncardia gastric cancer cases. Among these subjects, the difference in median survival between 2003‐2004 and 2011‐2012 varied from 0.1 to 0.2 years across all age groups. As shown in Table 5, the time at which 90% of the “uncured” patients are dead also had no significant change in each age group along with the calendar periods.

**Table 4:** Median survival time of “uncured” (years) for patients diagnosed with gastric cancer in Linzhou during 2003‐2012

Year	Age group (years)				Total
	19‐50	50‐59	60‐69	70‐99	
**Total**					
2003‐2004	1.0 (0.9‐1.1)	1.0 (0.9‐1.1)	0.9 (0.8‐1.0)	0.6 (0.6‐0.7)	0.9 (0.8‐0.9)
2005‐2006	1.1 (1.0‐1.3)	1.1 (1.1‐1.2)	1.0 (1.0‐1.1)	0.7 (0.7‐0.8)	1.0 (0.9‐1.1)
2007‐2008	1.2 (1.1‐1.4)	1.2 (1.1‐1.3)	1.1 (1.0‐1.1)	0.8 (0.7‐0.8)	1.0 (1.0‐1.1)
2009‐2010	1.2 (1.1‐1.3)	1.2 (1.1‐1.3)	1.1 (1.0‐1.2)	0.8 (0.7‐0.9)	1.1 (1.0‐1.1)
2011‐2012	1.1 (1.0‐1.3)	1.1 (1.0‐1.2)	1.0 (0.9‐1.1)	0.7 (0.6‐0.8)	0.9 (0.9‐1.0)
**Cardia gastric**					
2003‐2004	1.2 (1.0‐1.4)	1.2 (1.0‐1.3)	1.1 (0.9‐1.2)	0.7 (0.6‐0.8)	1.0 (0.9‐1.1)
2005‐2006	1.3 (1.1‐1.5)	1.3 (1.2‐1.4)	1.2 (1.0‐1.3)	0.8 (0.7‐0.9)	1.1 (1.0‐1.2)
2007‐2008	1.4 (1.2‐1.6)	1.3 (1.2‐1.4)	1.1 (1.0‐1.2)	0.8 (0.7‐0.9)	1.1 (1.0‐1.2)
2009‐2010	1.4 (1.2‐1.6)	1.4 (1.3‐1.5)	1.2 (1.1‐1.4)	0.9 (0.8‐1.0)	1.2 (1.1‐1.3)
2011‐2012	1.3 (1.1‐1.6)	1.2 (1.1‐1.4)	1.1 (1.0‐1.2)	0.8 (0.7‐0.9)	1.1 (1.0‐1.2)
**Noncardia gastric**					
2003‐2004	0.9 (0.7‐1.1)	0.9 (0.7‐1.0)	0.7 (0.6‐0.8)	0.6 (0.5‐0.7)	0.7 (0.6‐0.8)
2005‐2006	1.1 (0.8‐1.4)	1.0 (0.8‐1.2)	0.9 (0.7‐1.1)	0.6 (0.4‐0.8)	0.9 (0.7‐1.0)
2007‐2008	1.1 (0.9‐1.4)	1.1 (0.9‐1.3)	0.9 (0.7‐1.1)	0.8 (0.6‐0.9)	1.0 (0.8‐1.1)
2009‐2010	1.0 (0.7‐1.2)	1.0 (0.8‐1.2)	0.8 (0.6‐1.0)	0.7 (0.6‐0.9)	0.9 (0.7‐1.0)
2011‐2012	1.0 (0.7‐1.2)	1.0 (0.8‐1.2)	0.8 (0.6‐0.9)	0.7 (0.5‐0.8)	0.8 (0.7‐1.0)

**Table 5:** Time at which 90% of “uncured” are dead (year) for patients diagnosed with gastric cancer in Linzhou during 2003‐2012

Year	Age group (years)				
	Total	19‐50	50‐59	60‐69	70‐99
**Total**					
2003‐2004	3.1 (2.9‐3.2)	3.2 (3.0‐3.5)	3.3 (3.1‐3.5)	3.2 (3.0‐3.4)	2.3 (2.0‐2.5)
2005‐2006	3.2 (3.0‐3.3)	3.4 (3.1‐3.7)	3.5 (3.3‐3.7)	3.3 (3.1‐3.4)	2.5 (2.3‐2.7)
2007‐2008	3.4 (3.2‐3.5)	3.7 (3.5‐4.0)	3.7 (3.5‐3.9)	3.4 (3.2‐3.6)	2.7 (2.5‐2.9)
2009‐2010	3.5 (3.3‐3.6)	3.7 (3.5‐4.0)	3.8 (3.6‐3.9)	3.5 (3.3‐3.6)	2.9 (2.7‐2.9)
2011‐2012	3.4 (3.2‐3.6)	3.7 (3.5‐4.0)	3.7 (3.5‐3.9)	3.5 (3.3‐3.7)	2.8 (2.6‐3.1)
**Cardia gastric**					
2003‐2004	3.9 (3.2‐3.6)	3.6 (3.3‐4.0)	3.6 (3.4‐3.9)	3.5 (3.2‐3.7)	2.4 (2.1‐2.8)
2005‐2006	3.5 (3.3‐3.8)	3.7 (3.3‐4.1)	3.9 (3.7‐4.1)	3.6 (3.3‐3.8)	2.8 (2.4‐3.1)
2007‐2008	3.6 (3.4‐3.9)	4.0 (3.7‐4.3)	4.0 (3.7‐4.2)	3.6 (3.4‐3.9)	3.0 (2.7‐3.3)
2009‐2010	3.8 (3.6‐4.0)	4.1 (3.7‐4.4)	4.1 (3.9‐4.3)	3.8 (3.6‐4.1)	3.1 (2.8‐3.4)
2011‐2012	3.7 (3.5‐4.0)	4.1 (3.8‐4.4)	4.0 (3.7‐4.2)	3.8 (3.5‐4.1)	3.2 (2.9‐3.5)
**Noncardia gastric**					
2003‐2004	2.7 (2.3‐3.0)	3.0 (2.6‐3.5)	3.0 (2.5‐3.6)	2.7 (2.2‐3.1)	2.2 (1.7‐2.7)
2005‐2006	2.9 (2.5‐3.3)	3.4 (2.8‐3.9)	3.1 (2.6‐3.6)	3.1 (2.6‐3.5)	2.0 (1.3‐2.6)
2007‐2008	3.2 (2.8‐3.5)	3.5 (3.0‐4.0)	3.4 (3.1‐3.8)	3.1 (2.6‐3.5)	2.6 (2.1‐3.1)
2009‐2010	3.0 (2.7‐3.4)	3.3 (2.8‐3.7)	3.3 (2.9‐3.7)	2.9 (2.5‐3.4)	2.7 (2.2‐3.1)
2011‐2012	3.0 (2.7‐3.4)	3.3 (2.8‐3.8)	3.4 (3.0‐3.7)	3.0 (2.5‐3.4)	2.6 (2.2‐3.1)

## 4 DISCUSSION

To our knowledge, this is the first study that evaluated the proportion of gastric cancer patients who reached “statistical cure” in Linzhou population, which is valuable to healthcare planners, physicians, and patients. The gastric cancer five‐year relative survival rate was also estimated in our study, and it was seen to increase in each age group along with the calendar periods 2003‐2012. The cure fraction was observed to increase greatly over time periods; however, the median survival of uncured patients had no significant change. Therefore, the improvement in gastric cancer relative survival may be attributed to the increased cure fraction.

In our study, 20%‐40% of the patients diagnosed with gastric cancer during periods from 2003‐2004 to 2011‐2012 were estimated “cured.” The gastric cancer cure fraction was also evaluated in several studies in Japan and Europe countries. In Osaka, Japan, more than 50% of gastric cancer patients were estimated to be “cured” in 1996‐2000 through a population‐based study.^23^ It was much higher than results in our study, which may be due to earlier stage at diagnosis and improvement in treatment caused by the gastric cancer management.^23^ The cure proportions reported in Europe countries were approximately 20%‐30%, which were lower than those in Japan and recent period in our study.^24^, ^25^, ^26^, ^27^, ^28^ The difference in cure proportion usually related to the gastric cancer treatment and stage at diagnosis.^12^, ^23^ East Asia countries including Japan and China had large gastric cancer burden, and population‐based cancer screening program was carried out nationwide or in high‐risk areas; however, that is not the case in Europe countries.

The interpretation of trends in cure proportion should also consider survival time of the “uncured”.^12^ Higher cure proportion and longer survival time of the “uncured” were interpreted as a general improvement in treatment, and higher cure proportion with shorter time of “uncured” was interpreted as a selective improvement in treatment, which enables uncured patients with a longer survival time to be cured. However, higher cure proportion with unchanged survival time of the uncured might occur following the introduction of some diagnostic procedure, such as screening, which included more patients who have no excess risk.^12^, ^13^ Besides the high cure proportion in Linzhou, the survival time of “uncured” patients hardly changed along with calendar periods. Hence, the improvement in cure proportion may be related to the introduction of screening program.

Linzhou was known to have a large disease burden of upper gastrointestinal cancer including gastric cancer and esophageal cancer. National programs to control disease burden were conducted, so local doctors have more access to be trained by professionals from national cancer centers that treat patients with advanced treatment. The estimated gastric cancer relative survival in Linzhou was 33% among patients diagnosed during 2003‐2012 with highest value of 44% in most recent period, which was higher than that in the national level estimated using data from 17 cancer registries in China.^29^ Shanghai reported the 5‐year relative survival of 30% during 2002‐2003 using population‐based cancer registration data.^30^ Interpretation of difference in gastric cancer survival should take into account the differences in health awareness and early detection, and the availability, development of, and accessibility to cancer treatment within the population.^29^ An organized screening program for gastric cancer was initiated among the target population who aged 40‐69 years since 2005 in Linzhou city, and a total of 22,869 subjects were screened at the end of the calendar year 2012. Endoscopy with indicative biopsy was used to detect and diagnose gastric cancer and precancerous lesions, and participants were recalled to the clinic when early lesions were histologically diagnosed; then, intervention methods appropriate to the lesions' severity were used.^3^ Hence, higher proportion of early‐stage gastric cancer and precancerous cases were detected and treated, thereby improving the survival of gastric cancer. The increasing trend of gastric cancer survival and its high value of relative survival are related to screening; however, the lead time bias and incomplete follow‐up could not be ruled out.

In recent years, the gastric cancer treatment method advanced not only in early‐stage cancer including laparoscopic and open surgical resection^31^, ^32^ but also in advanced‐stage cancer including preoperative and postoperative radiotherapy.^33^ The screening program carried out provided training of early gastric cancer identity, diagnosis, and treatment each year for local professionals by national experts.^5^ In this study, cure fraction was seen to increase largely within the calendar period, and it was not caused by the effect of lead time bias due to the advantage of cure model.^13^, ^14^ Therefore, the effect of the carried out gastric cancer screening may play a role in the improved cure fraction; however, the role of improvement in radiology and/or surgery could not be ruled out.

Cure models bring researchers more information of patients' survival; however, limitations should be considered when interpreting the results. Firstly, it would output the estimation of cure fraction in cure model no matter whether the statistical cure is reached or not. In our study, the cumulative relative gastric cancer survival curve reached the plateau after five years from the diagnosed date, which suggested that statistical cure was reached at that time. Secondly, analysis stratified by gastric cancer stages could not be conducted due to the lack of cancer stage variable, which was not mandatory in Linzhou Cancer Registry. Thirdly, our study could not evaluate the impact of overdiagnosis of gastric cancer screening on the estimated cure fraction; however, the impact was much lower in cancers with poor prognosis than in cancers with good prognosis such as breast cancer, thyroid cancer, and prostate cancer.

In conclusion, improved relative survival and cure fraction during 2003‐2012 were observed in Linzhou, and it was mainly caused by the widespread of the organized gastric cancer screening.

## CONFLICTS OF INTERESTS

The authors declared no potential conflicts of interest with respect to the research, authorship, and/or publication of this article.

## Data Availability

The data that support the findings of this study are available on request from the corresponding author. The data are not publicly available due to privacy or ethical restrictions.
